# Minerals in biology and medicine

**DOI:** 10.1039/d0ra09992a

**Published:** 2021-01-06

**Authors:** Oliver W. L. Carter, Yingjian Xu, Peter J. Sadler

**Affiliations:** Department of Chemistry, University of Warwick Gibbet Hill Road Coventry CV4 7AL UK P.J.Sadler@Warwick.ac.uk; MAS CDT, Senate House, University of Warwick Coventry CV4 7AL UK; GoldenKeys High-Tech Materials Co., Ltd, Building B, Innovation & Entrepreneurship Park Guian New Area Guizhou Province 550025 China goldenkeys9996@thegoldenkeys.com.cn

## Abstract

Natural minerals (‘stone drugs’) have been used in traditional Chinese medicines for over 2000 years, but there is potential for modern-day use of inorganic minerals to combat viral infections, antimicrobial resistance, and for other areas in need of new therapies and diagnostic aids. Metal and mineral surfaces on scales from milli-to nanometres, either natural or synthetic, are patterned or can be modified with hydrophilic/hydrophobic and ionic/covalent target-recognition sites. They introduce new strategies for medical applications. Such surfaces have novel properties compared to single metal centres. Moreover, 3D mineral particles (including hybrid organo-minerals) can have reactive cavities, and some minerals have dynamic movement of metal ions, anions, and other molecules within their structures. Minerals have a unique ability to interact with viruses, microbes and macro-biomolecules through multipoint ionic and/or non-covalent contacts, with potential for novel applications in therapy and biotechnology. Investigations of mineral deposits in biology, with their often inherent heterogeneity and tendency to become chemically-modified on isolation, are highly challenging, but new methods for their study, including in intact tissues, hold promise for future advances.

## Abstract 2

Minerals play important natural roles in biology and offer relatively unexplored potential for the design of novel drugs, for the diagnosis of disease, and for discovery of new biotechnological applications *via* multisite recognition of biomolecules.

## Graphical abstract

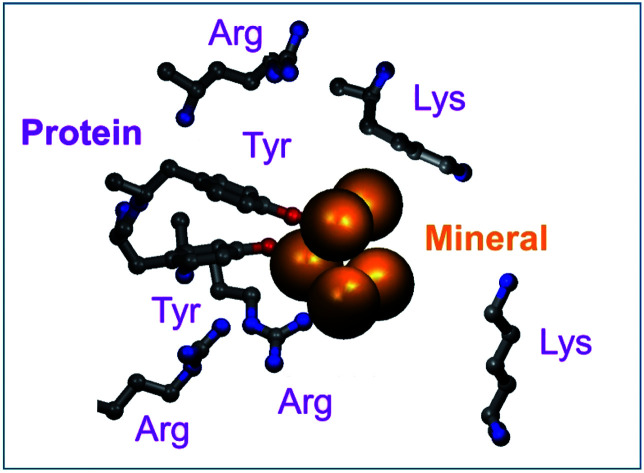

## 1. Introduction

Until recently, it was commonly believed that the chemistry of life is organic chemistry, and that inorganic chemistry (mineral chemistry) is confined to the inanimate world. Nowadays it is clear that although the chemistry of carbon is of course crucial, at least 18 other elements are essential for mammalian life.^1^ For several metals, the genetic codes for proteins which control their absorption, transport, distribution and excretion from the body are becoming well understood. Remarkably, 10% of the expressed genes in the human genome code for zinc proteins, with zinc playing a key role in transcription factors and a wide range of proteins and enzymes. Other essential trace d-block metals with vital roles in enzymes include Mn, Fe, Co, Cu and Mo, along with the more mobile bulk alkali and alkaline earth metals Na, K, Mg and Ca. The latter have key roles in the transmission of nervous impulses, membrane potentials, muscle contraction, energy metabolism, protein synthesis, cell replication, skeletal structures, and many other biochemical pathways. Whether it will be possible to recognise genetic codes for all the essential elements is an intriguing question. Probably it will not, bearing in mind that proteins are never totally selective for particular metal ions.

Here we focus on the roles of minerals in biology and medicine. We use selected examples to highlight both the natural roles of minerals (*e.g.* in bones, teeth, balance organ in the inner ear), their use in medicine (*e.g.* phosphate binding, ion exchange), in biotechnology (*e.g.* extraction and purification of DNA), as well as the potential for the discovery of new medicines based on novel target recognition mechanisms. Within the concept of multi-site recognition of targets, we also discuss oligomeric metal complexes and metallopolymers.

Knowledge of natural biomineralisation processes has led to laboratory procedures using organic templates to fashion nanominerals. The mechanisms of inorganic morphosynthesis have been summarised by Mann and Ozin: deposition on patterned materials (preorganised organic architectures and templates), co-assembly of inorganic precursors, organic molecules and aggregates (*e.g.* micelles), metamorphic reconstruction, and microphase separation.^2^ Such mineral engineering has much potential for the design of novel materials for a variety of uses.^3^

This article was written during lockdown phases of COVID19, a period during which the urgent need to discover new medicines, especially for treatment of viral infections, became even more evident.^4^

## 2. Natural Chinese mineral medicines – ‘stone drugs’

Although the potential for the use of minerals in drug design has yet to be widely explored in modern pharmacology, the medicinal value of minerals and other ‘stone drugs’ has been recognised in China for >2000 years.^5^ Indeed, the use of minerals and other natural substances is central to Traditional Chinese Medicine (TCM). The Chinese scientist Tu Youyou, winner of the 2015 Nobel Prize for Physiology or Medicine for her discovery of the antimalarial artemisinin isolated from a herb, is a Professor at the China Academy of Traditional Chinese Medicine.

**Fig. 1:**
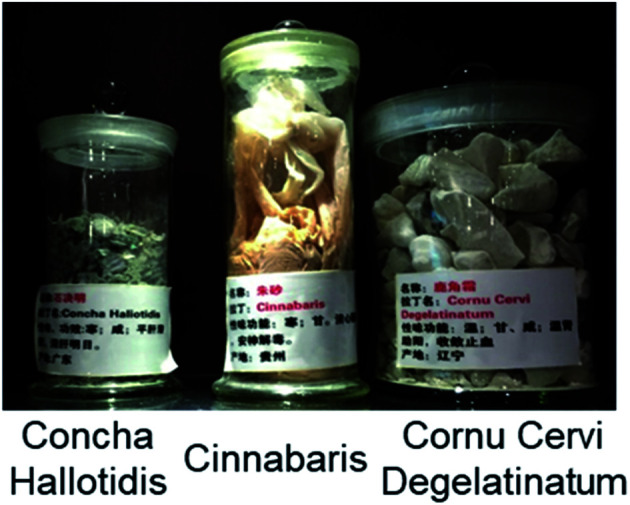
Examples of Chinese mineral drugs on display at Guizhou University of Traditional Chinese Medicine (photographed in October 2018). *Concha Hallotidis* (abalone shell) is predominantly microscopic calcium carbonate. Cinnabaris (cinnabar) is mercuric sulphide (HgS), and *Cornu Cervi Degelatinatum* from animal horns is rich in calcium phosphate.

Chinese universities with programs in TCM often house extensive collections of mineral drugs. A few examples from Guizhou University of Traditional Chinese Medicine are shown in Fig. 1.

**Fig. 2:**
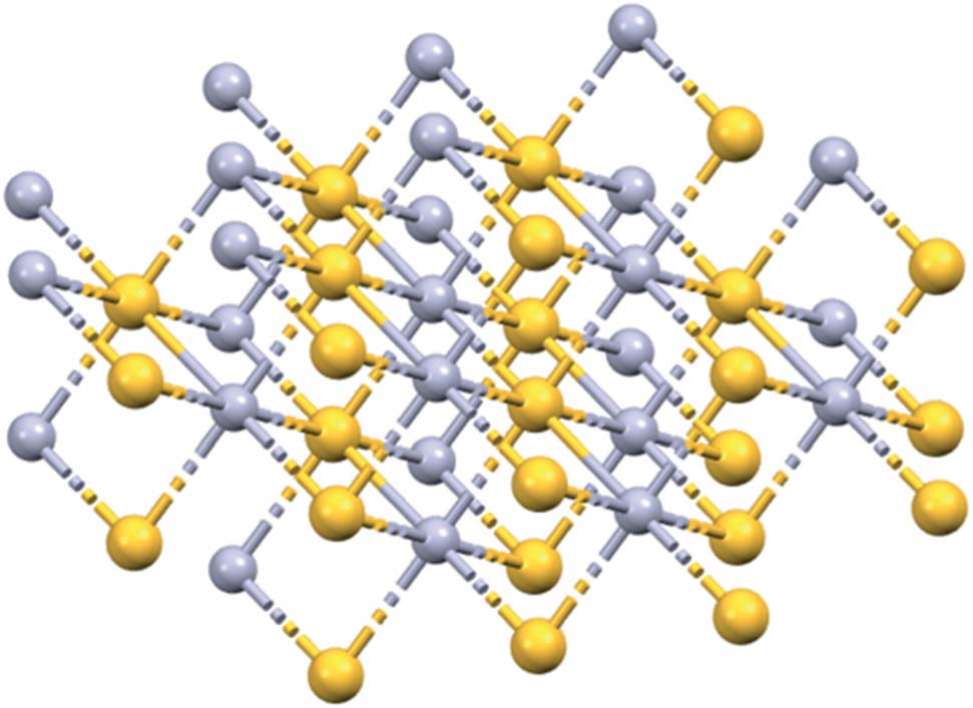
X-ray crystal structure of HgS (Cinnabar, mp 853 K). Hg, mauve; S, yellow. Hg–S, 2.791 Å; Hg–Hg, 3.947 Å. S–S, 3.947 Å. OSTI identifier: 1187346. DOI: 10.17188/1187346.

*Concha Hallotidis* (abalone shell) consists of predominantly microscopic calcium carbonate (usually calcite and aragonite) tiles stacked like bricks interwoven with a small amount (<*ca.* 5% by weight) of organic shell-matrix proteins. Within the tiles, proteins are thought to play important roles in controlling the synthesis and structure of the mineral component.^6,7^ Cinnabar (mercuric sulphide, HgS, Fig. 2) is said to be used in about 40 traditional Chinese medicines today.^8^ HgS is relatively insoluble, poorly absorbed, and has a low toxicity. It has been suggested that the mechanism of cinnabaris toxicity is due to oxidative damage and induction of apoptosis, although studies into its neurotoxicity require futher work.^9^*Cornu Cervi Degelatinatum* from animal horns is rich in calcium phosphate and is an ingredient in “Bushen Guchong Wan” – a combination of Chinese medicines used in the treatment of gynaecological conditions.^10,11^ In general, elucidating the mechanisms of action of minerals used in TCMs is challenging, and warrants further research now that powerful techniques are available for studying their composition and structure, including dynamic exchange and release of both metal ions and ligands from both the surface and bulk mineral, especially in biological media, and in the presence of various cell types.

**Fig. 3:**
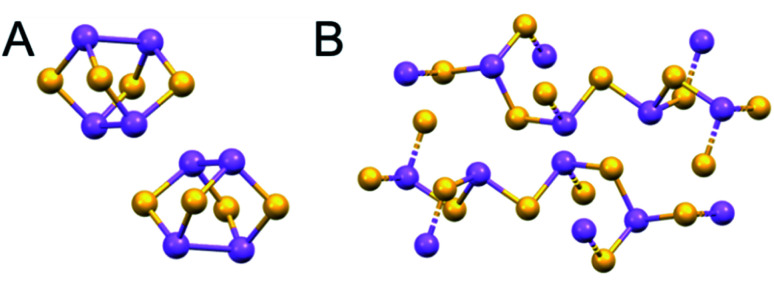
X-ray crystal structures of naturally-occurring arsenic sulfide minerals (A) Realgar, As_4_S_4_, (mp-542846) and (B) Arsenicum, As_2_S_3_ (mp-641). As, purple; S, yellow. (mp-542846 DOI: 10.17188/1266759).^15^

The use of arsenic compounds in TCM includes Arsenicum, arsenic's chief ore (As_2_S_3_, golden colour, ‘yellow orpiment’), and Realgar (As_4_S_4_, red arsenic).^12,13^ Recent work has also indicated the potential of these arsenic compounds as anticancer treatments.^14^ These structures contain bridging sulfides (Fig. 3).

There is current TCM interest in the medicinal properties of orchid (*Dendrobium*) plants, which are being used in pre-workout supplements to boost physical and athletic performance. Some are rich in d-block metals (Ni, Zn, Fe, Cr, Ti) and in lanthanides.^16^

## 3. Metal nanoparticles and bio-active minerals

### 3.1. Atom-by-atom synthesis of metal nanoparticles

**Fig. 4:**
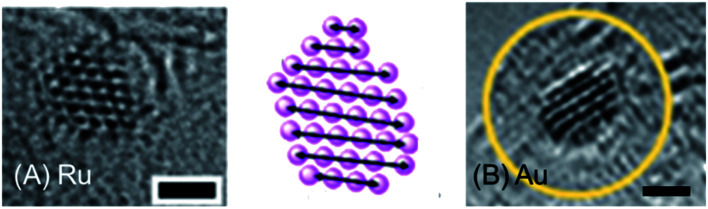
Ruthenium and gold nanocrystals fabricated on a graphitic support by electron bombardment of Ru(ii) and Au(iii) complexes encapsulated in polymer micelles deposited on a lacey carbon EM grid in an aberration-corrected high-resolution transmission electron microscope. Interatomic distances: Ru–Ru 2.48 Å, Au–Au 2.73 Å. Scale bars 1 nm. Adapted from ref. 20 with permission from the Royal Society of Chemistry, copyright 2016.^20^

The sizes of metal nanocrystals can be precisely controlled by synthesis using metal complexes encapsulated in polymer micelles supported on a grid in the beam of a transmission electron microscope (Fig. 4).^17^ For example, the beam can reduce Au(iii) to Au(0) and generate crystals ranging from just a few gold atoms to many hundreds on a graphitic matrix. This procedure can readily be applied to other metals including Os, Ir, and Ru, and also generate nanoalloys. Such nanocrystals might provide centres for trapping and identification of micro-organisms and viruses, either as zero oxidation-state metals, or conjugated to surface vectors. Surface metal atoms may oxidise readily on exposure to oxygen or other agents.^18^ Such nanocrystals can also be generated by laser or microwave irradiation.^19^

Interestingly, breakfast cereals such as cornflakes are often fortified with micro-crystals of metallic iron (body-centred cubic alpha-Fe) which dissolves slowly at stomach pH, allowing some of the iron to be absorbed into the body.^21^

### 3.2. Colloidal gold – surface reactivity and photothermal therapy

Gold nanoparticles have been known since about 1650 when Andreas Cassius prepared intensely coloured colloidal gold sols. The colours of the sols depend on particle size; red sols contain smaller particles (<100 nm diameter) than blue sols. They are easily prepared with diameters of 5 to 250 nm by reduction of Au(iii) (*e.g.* [AuCl_4_]^−^, Au dissolved in aqua regia) with *e.g.* citrate or Sn(ii). Gold nanoparticles have localized surface plasmon resonances, which cause optical field enhancements (*e.g.* Raman and fluorescence).^22^

The surface of colloidal Au(0) nanoparticles is often partially oxidized to Au(i), and is usually covered with a layer of anions such as citrate.^22^ Surface covering prevents aggregation of the particles. Au(i) has a high specific affinity for thiolate sulfur, so thiolate ligands are widely used to modify the surface. Modified gold surfaces can be coated with proteins, *e.g.* antibodies which target cell surface receptors, and are widely used in immuno-histochemistry.^22^

DNA-coated-Au nanoparticles are readily taken up by cells. A HeLa cell can take up *ca.* 6000 citrate-coated Au nanoparticles.^23^ This may involve protein-coating from the culture medium since the cell membrane is negatively charged. There is medical interest in the use of gold nanoparticles as gold nanorods in photothermal therapy with near infrared light activation (800–1200 nm).^24^

### 3.3. Hydroxyapatite – bones and teeth

There are natural calcium phosphate minerals in the body. Seventy percent of bone is the Ca^2+^ mineral hydroxyapatite, Ca_10_(PO_4_)_6_(OH)_2_. The mineralisation is carefully controlled by special cells, osteoblasts and osteocytes, in the presence of collagen fibres and a few other proteins.^25^ Knowledge of the chemistry of bone has inspired the chemical synthesis of hybrid materials for the regeneration of human bone and dental tissues.^26^*Cornu Cervi Degelatinatum*, deer horn (antlers, Fig. 1), are made of bone and can regenerate.^27^ Mineralized scaffolds for bone regeneration containing not only calcium and phosphate, but also trace elements such as iron and manganese, are being constructed in the laboratory.^26,28^

**Fig. 5:**
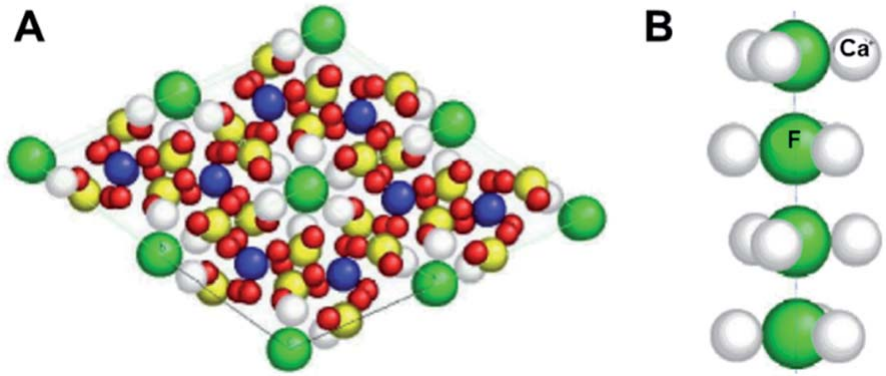
X-ray crystal structure of fluorapatite, the phosphate mineral Ca_5_(PO_4_)_3_F, showing (A) 2 × 2 unit cells, and (B) a column of F^−^ ions. Each F^−^ ion is bonded to F^−^ ions above and below it (F–F 3.44 Å) and to 3 Ca^2+^ ions (F–Ca 2.463 Å) which form a triangle around it. Ca, grey; P, yellow; O, red; F, green. (A) is reproduced and (B) adapted from ref. 29 with permission from AO Research Institute Davos, copyright 2001.^29^

Hydroxyapatite is the major (96%) constituent of hard tooth enamel. Toothpastes often contain Sr^2+^ which can substitute for Ca^2+^, and fluoride which can replace hydroxide ions in the enamel. The structure of fluorapatite (Ca_5_(PO_4_)_3_F) is shown in Fig. 5. Chloride and carbonate substitutions can also occur. In general, the heterogeneous nature of such minerals is notable for conferring an ability to modify their structural and functional properties (often reversibly).

The surface properties of hydroxyapatite can be used as a medium for the chromatographic separation of proteins.^30^ The surface has negatively-charged sites (phosphates) at basic pH, and positively-charged sites (Ca^2+^) at acidic pH values when the phosphates are partially protonated.^31^ Hence the separation characteristics can be adjusted according to the charge on the protein.

Another important use of hydroxyapatite chromatography is in the fractionation and recovery of ssDNA, dsDNA and RNA viral nucleic acids from mixed viral assemblages.^32,33^ Elution of these different forms depends on the extent of interaction of backbone nucleotide phosphates with the hydroxyapatite Ca^2+^ ions, and can be controlled by the phosphate concentration in the buffer (0–0.4 M).

### 3.4. Calcium carbonate – balance organ

**Fig. 6:**
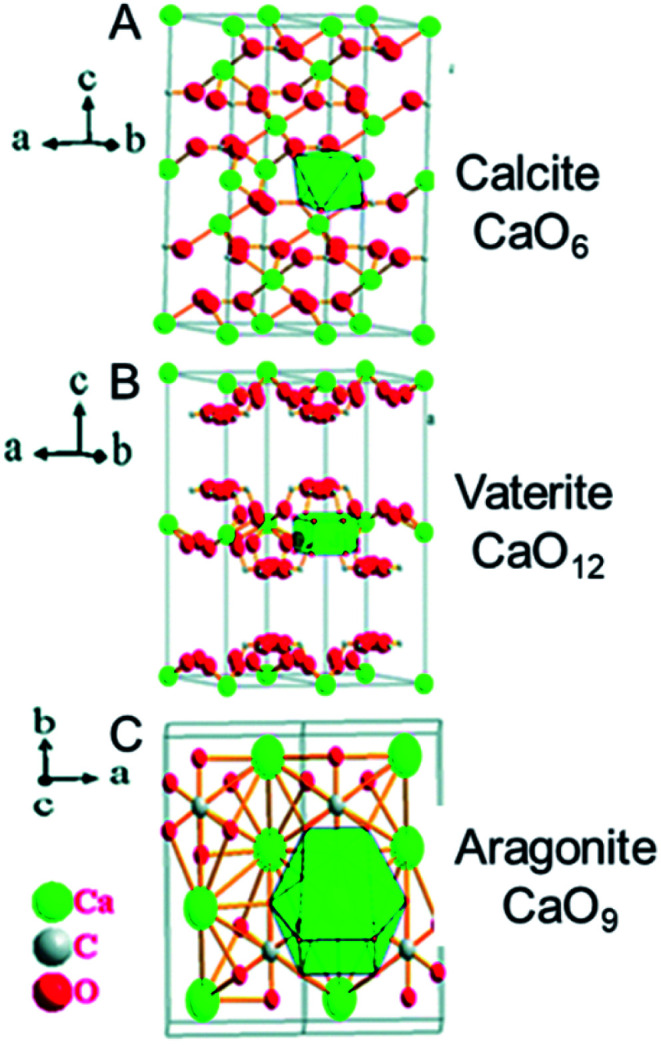
X-ray crystal structures of calcium carbonate, CaCO_3_. (A) Calcite, (B) vaterite, and (C) aragonite. The coordination number of Ca^2+^ increases from 6 in calcite, to 9 in aragonite and 12 in vaterite (indicated on the right), which is accompanied by an increase in ligand repulsion, and a decrease in stability. The most stable form, calcite, is found in the human ear. Reproduced from ref. 35 with permission from the Royal Society of Chemistry, copyright 2017.^35^

The organ in the inner ear that senses balance and movement contains micron-sized crystals of calcium carbonate, CaCO_3_, in a gelatinous protein matrix. There are 3 crystal forms of calcium carbonate with different packing of the Ca^2+^ cations and CO_3_^2−^ anions in the lattice: aragonite, vaterite, and calcite (Fig. 6). In the balance organs of mammals, it is usually calcite, however it is aragonite in amphibians.^34^

An interesting property of inorganic minerals is their ability to contain defects such as dislocations, substitutions and interstitial inclusions. This potential for heterogeneity of biominerals could be exploited more widely in drug design, to provide, for example, synergistic slow release of metal ions and anions.

### 3.5. Silicate minerals

#### 3.5.1. Oligonucleotide separation on mica

Mica consists of hydrous potassium aluminium silicate, and the term covers about 30 members. Its layer structure with negatively-charged silicate sheets has a wide range of possible compositions: X_2_Y_4−6_Z_8_O_20_(OH,F)_4_, where commonly X = K, Na, or Ca, Y = Al, Mg or Fe, and Z = Si or Al. Mica can be cleaved almost perfectly to give flat surfaces, useful as supports for molecules which can be studied by *e.g.* various types of microscopy.

**Fig. 7:**
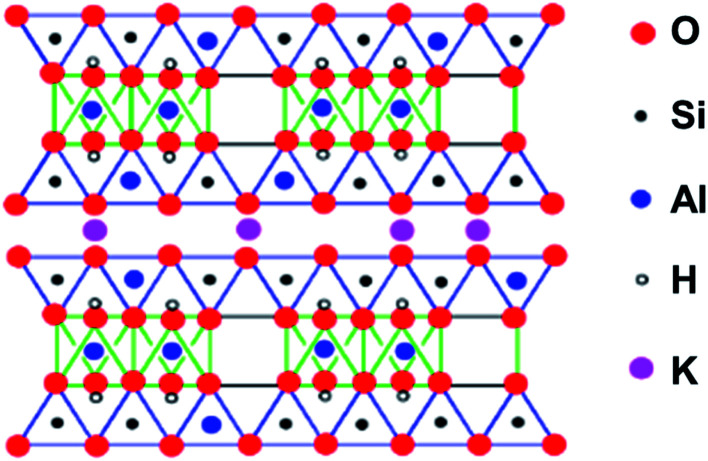
The structure of a mica, muscovite, (KF)_2_(Al_2_O_3_)_3_(SiO_2_)_6_(H_2_O). The space between the layers is occupied only by K^+^ ions. The layer structure facilitates cleavage to produce flat surfaces. Adapted from https://employees.csbsju.edu/cschaller/PrinciplesChem/network/NWalumina.htm (accessed 17^th^ November 2020), courtesy of Chris Schaller.

**Fig. 8:**
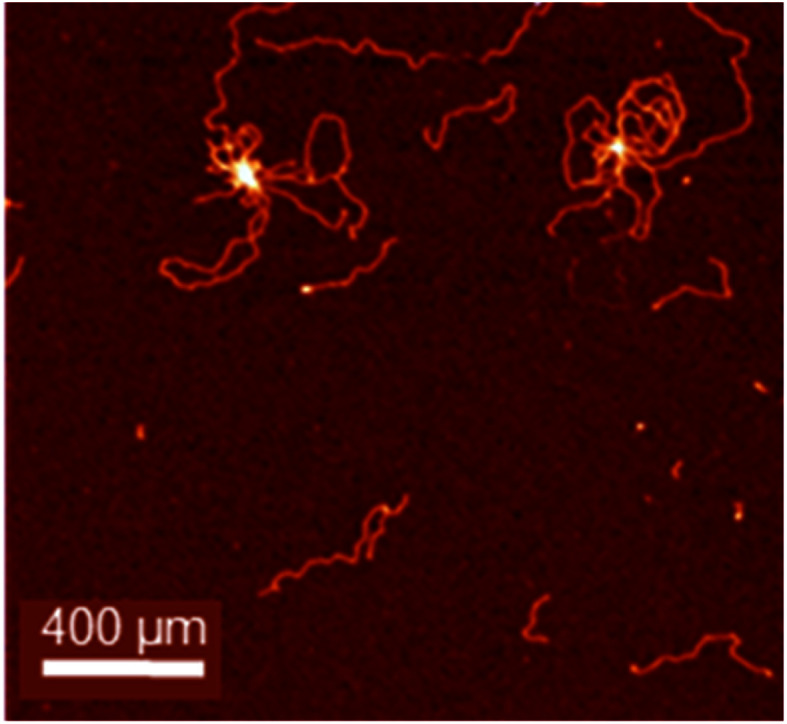
pBR322 DNA plasmid deposited onto freshly cleaved mica treated with Mg^2+^ to ensure adsorption of the negatively-charged DNA. DNA condensation has been induced by the tetranuclear complex [Os_4_(η^6^-*p*-cymene)_4_(μ^2^-OH)_4_(pyrazine)_2_]^4+^. Larger loops of DNA can also be seen. Adapted from ref. 37 with permission from Wiley-VCH, copyright 2016.^37^

As an example, a diagrammatic structure of muscovite mica is illustrated in Fig. 7. Importantly, the flat surface can be modified to change the binding properties. Typically, for negatively charged DNA or RNA immobilization, the surface can be coated with cations, *e.g.* Mg^2+^, Ca^2+^, Co^2+^, Ni^2+^, or Zn^2+^, allowing imaging, *e.g.* by AFM (Fig. 8).^36^

#### 3.5.2. Toxicity of asbestos

**Fig. 9:**
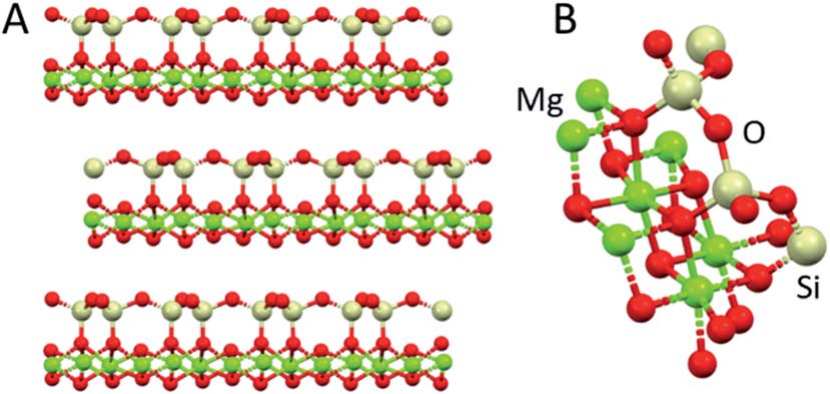
X-ray structure of chrysotile, Mg_3_(Si_2_O_5_)(OH)_4_. (A) Layer structure, and (B) half of the unit cell (ICSD 41363). Mg, green; O, red; Si, cream.

The six naturally occurring silicate minerals known as asbestos are useful building materials, being long and thin fibrous crystals, which are electrical and acoustic insulators and heat-resistant. Their structures are based on anionic SiO_4_ tetrahedra arranged into sheets and chains with bound Mg^2+^, Al^3+^, Ca^2+^, Fe^2+/3+^, Mg^2+^, K^+^ and Na^+^ cations. However, asbestos is toxic. The associations between asbestos exposure, lung cancer, and mesothelioma are well established.^38^ There is concern that talc, a hydrous magnesium silicate Mg_3_Si_4_O_10_(OH)_2_ and widely used cosmetic product, with a related but different structure, can be contaminated with asbestos.^39^ The most common form of asbestos, chrysotile (Mg_3_(Si_2_O_5_)(OH)_4_), Fig. 9, contains layered SiO_4_ and octahedral Mg^2+^ sheets.^40^ Some Mg^2+^ ions are replaced by Fe^2+/3+^ and other cations. The presence of redox-active iron may play a role in the toxicity *via* oxidative damage to cells. The surface charge of asbestos fibres allows them to adsorb on cellular macromolecules such as proteins, including cell surface proteins, DNA and RNA.^41^

### 3.6. Toxicity of nickel subsulfide

Nickel and its compounds are considered the most common metal-based contact allergens in the industrialised world, causing type I and IV (delayed) hypersensitivity, involving the interaction of T-cells, monocytes, and macrophages. Several sparingly soluble and crystalline nickel compounds are toxic, primarily by reducing levels of glutathione and binding to protein sulfhydryl groups.^42^ Of particular concern is nickel subsulfide, Ni_3_S_2_, which is carcinogenic.^43^ It is used in the manufacture of Li batteries and a component in the refinement of some Ni ores.^44^ Ni_3_S_2_ particles can bind to mammalian cell surfaces and are readily taken up. Once in cells, Ni ions are released and attack proteins and DNA.^43^

### 3.7. Lanthanum carbonate – oral drug

**Fig. 10:**
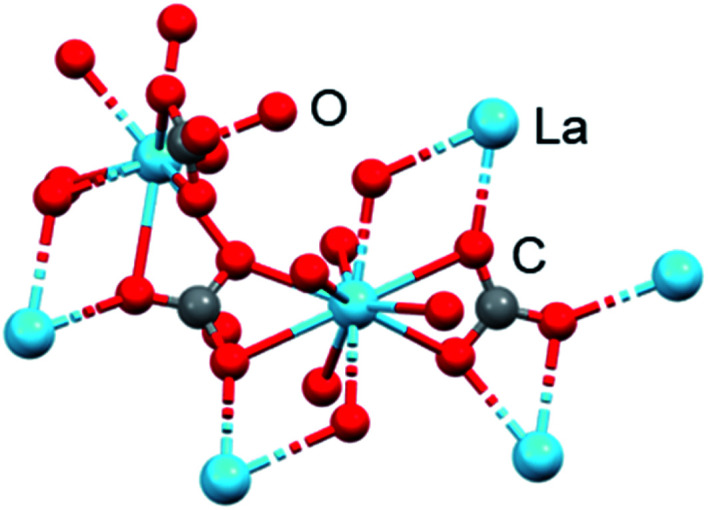
X-ray crystal structure of La_2_(CO_3_)_3_·8H_2_O (CCDC1599003). La^3+^ is 10-coordinate with coordination sites occupied by water, bidentate and monodentate carbonate, La–O bond lengths 2.52–2.74 Å. It is a layer structure with alternate rows of carbonate anions and lanthanide cations. A quarter of the water molecules are unbound and between layers. La, blue; O, red; C, grey.^49^ (ICSD-22224).

Lanthanide(III) ions are all of similar size, with the well-known contraction in ionic radius across the series for 6-coordinate ions from 1.03 Å for La^3+^ to 0.86 Å for Lu^3+^. They have a ‘hard’ character, with strong ionic character in the bonds, and a strong affinity for oxygen ligands. Such large ions can readily accommodate high coordination numbers of 9, even 10. This can be seen in Fig. 10, in which both water and carbonate oxygens are bound to La^3+^ in lanthanum carbonate, La_2_(CO_3_)_3_·8H_2_O, a relatively insoluble mineral.

Lanthanum minerals are probably best known for their use in commercial solid oxide fuel cells and high temperature superconductors.^45,46^ Lanthanum carbonate (Fig. 10, trade name Fosrenol) is now an established drug, approved for removal of phosphate from the body in cases of hyperphosphatemia, which restricts calcium absorption, *e.g.* in chronic renal failure. It is administered orally at doses of 375–3000 mg La per day.^47^ Oral doses of up to 4.7 g per day were found to be well tolerated in a clinical trial.^48^

This use of lanthanum carbonate relies on the strong affinity of La^3+^ for oxygen ligands from both carbonate and phosphate, and its insolubility in aqueous media even under acidic conditions. The application as a phosphate-removal drug was originally developed by Murrer and colleagues at Johnson Matthey.^50^ Lanthanum(iii) binds phosphate in the physiologically relevant pH range of 3 to 7. In simulated gastric fluid, La^3+^ binds approximately 97% of the available phosphate at pH 3–5 and 67% at pH 7, when it is present in a two-fold molar excess over phosphate.

### 3.8. Zirconium oligomers and minerals – antiperspirants and potassium therapy

The ionic radius of 6-coordinate Zr(iv) of 0.86 Å is similar to that of Lu(iii). With its high charge, coordination numbers of up to 8, and polarising power, bound water is highly acidic and readily deprotonates to give hydroxide and oxide ligands which can bridge to other Zr(iv) ions forming oligomers, even in acidic solutions. Such polymers are often used as antiperspirants, forming a coating on the skin, blocking sweat pores, and preventing the escape of odours (often caused by bacteria).^51^ Aluminium(iii) forms similar hydroxyl/oxo polymers. An example of such an antiperspirant is the so-called aluminium zirconium tetrachlorohydrex glycine complex.^52^ Aluminium Chloro Hydrate (ACH), another antiperspirant, can form polycationic species such as εAl_13_ and εAl_30_ with molecular weights reaching over 5000 Da in solutions around pH 4.^53^

**Fig. 11:**
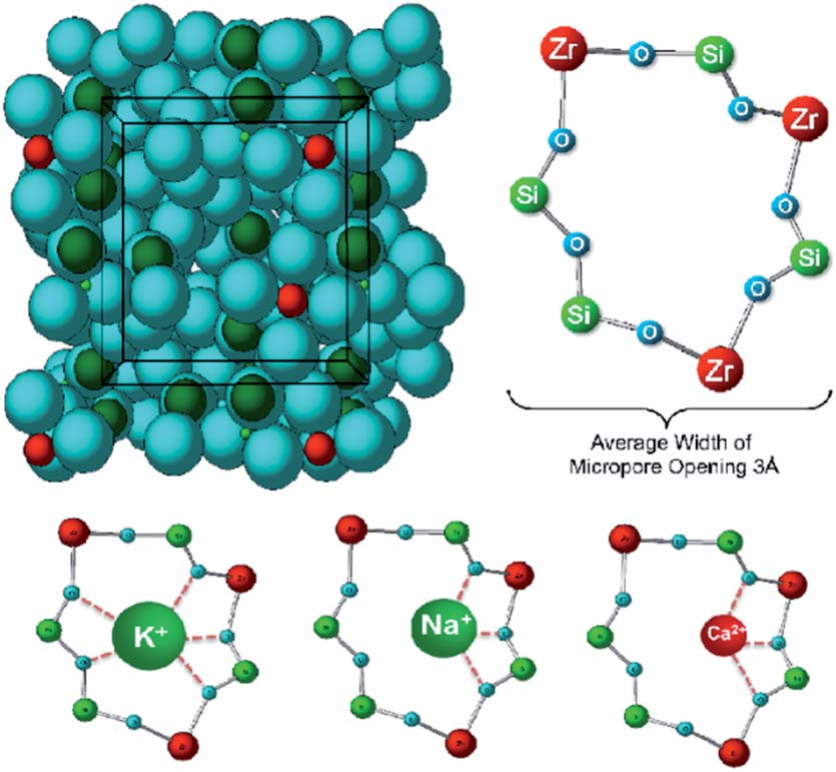
Crystal structure of sodium zirconium cyclosilicate, (2Na·H_2_O·3H_4_SiO_4_·H_4_ZrO_6_)_*n*_ (ZS-9). The oral drug Lokelma, used to lower K^+^ levels in the body. It is a cation exchanger and contains pores which bind cations, especially K^+^. The pores resemble those in the physiological K^+^ channel. Blue, O; red, Zr; green, Si (except where labelled in bottom row). Reproduced from ref. 55 with permission from Public Library of Science, copyright 2014.^55^

Sodium zirconium cyclosilicate (ZS-9) is a non-absorbed cation exchanger that selectively binds potassium in the intestine, and used for treatment of chronic hyperkalaemia at doses of 2.5–10 g per day administered with meals.^54^ It is a non-absorbable mineral which selectively binds K^+^ (and NH_4_^+^) in exchange for Na^+^ and H^+^ in the whole gastrointestinal tract.^55^ There are pores in the structure which match well the size of K^+^ and bind it with high affinity (Fig. 11).^54^

### 3.9. Metallopolymers

In recent years there have been significant advances in the design of synthetic polymers with a wide range of composition of organic backbones (*e.g.* block co-polymers) and 3D architectures.^56,57^ Such polymers can offer a range of useful new features in drug design and disease diagnosis. For example, their shapes and the properties of attached functional groups may change in response to pH changes, temperature change, and attack by redox agents or specific enzymes. Moreover, their backbones may be decorated to provide targeting and metal-binding ligands, *e.g.* chelating ligands such as nitrilotriacetate and terpyridine. There are extensive reports of incorporation of Fe(ii), Co(ii), Ti(iv) and Zr(iv) cyclopentadienyl centres on account of their activity in redox reactions and their catalytic properties. Importantly, they could be designed to provide multipoint recognition sites for macromolecular biological targets including cell surfaces.

Such nanoparticles (*e.g.* 50–300 nm diameter) may also accumulate more in tumours compared to normal tissue, providing *e.g.* selective drug delivery, according to the Enhanced Permeability and Retention (EPR) effect, although evidence for this in the body is debated.^58^ As an example in drug delivery, cyclic peptides can be conjugated to the biocompatible polymer poly(2-hydroxypropylmethacrylamide) (pHPMA).^59^ The conjugates were functionalized with organoiridium anticancer complexes. They self-assemble into elongated cylindrical shapes. Interestingly, the drug-loaded nanotubes exhibited more potent antiproliferative activity toward human cancer cells than either free drug or the drug-loaded polymers. The nanotubes themselves were nontoxic. The increased potency of the conjugate appeared to be related to a more efficient mode of action rather than higher cellular accumulation of iridium.

The flexibility in the structures of many polymers makes them attractive for adopting unusual shapes, *e.g.* concave surfaces which can recognise spherical viruses. Such a strategy is being explored using molecularly imprinted polymers (MIPs), materials that act as synthetic antibodies, an example being virus-neutralizing hydrogel-based MIPs.^60^

## 4. Viruses and microbes

The genetic material of virus particles is contained in an inner core, and is usually either single-stranded or double-stranded RNA (*e.g.* cold, influenza, SARS, COVID-19, hepatitis C, hepatitis E, West Nile fever, Ebola virus disease, rabies, polio and measles, tomato mosaic viruses) or DNA (*e.g.* smallpox, herpes, and the chickenpox viruses).^61^

**Fig. 12:**
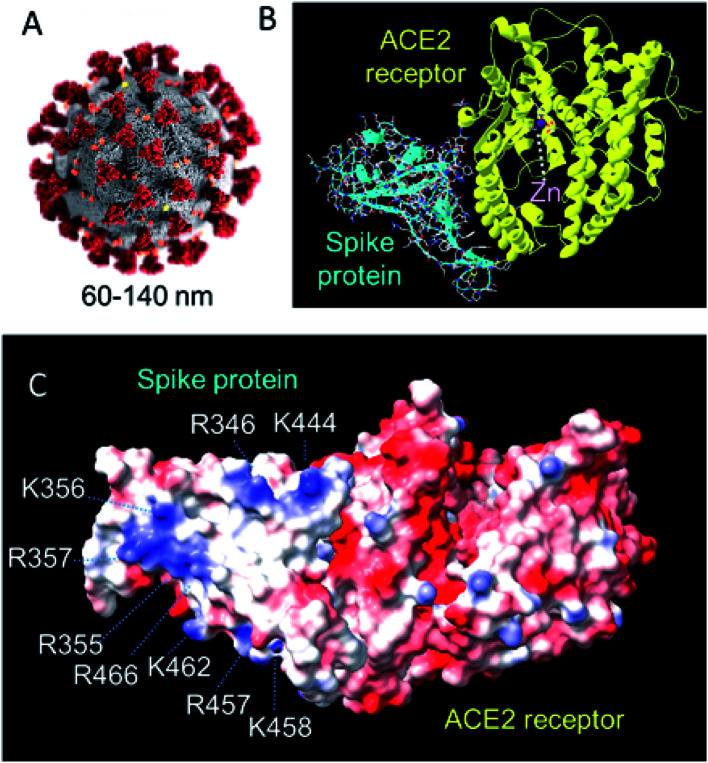
(A) Structure of SARS-CoV-2. The virus has a diameter of *ca.* 60–140 nm with 9–12 nm crown-like spikes of the transmembrane spike (S) glycoprotein on its surface.^64^ This S protein forms trimers and facilitates binding to angiotensin-converting 2 (ACE2), a zinc enzyme on host respiratory tract cells.^65–68^ Reproduced with permission from CDC/Alissa Eckert, MSMI; Dan Higgins, MAMS.^69^ (B) The 2.5 Å resolution X-ray crystal structure of coronavirus spike receptor-binding domain (largely β-sheet) complexed with its receptor ACE2 (largely α-helical, based on pdb 6lzg, courtesy of Claudia Blindauer). ACE2 is a protease. The catalytic active-site Zn(ii) (coordinated to 2His, Glu and H_2_O) is labelled. 13 polar residues are involved in hydrogen bonds and/or salt bridges with the SARS-CoV-2. (C) Surface electrostatic charge distribution in the structure shown on B (red, −ve; blue, +ve). The glycan chains are not defined in this structure. Notable is the cluster of positively-charged Lys and Arg residues on this face of the spike protein that might be a target for recognition by *e.g.* negatively charged POMs. (pdb 6lzg, prepared using UCSF ChimeraX).

The SARS-CoV-2 virus (responsible for the COVID-19 pandemic) contains single-stranded RNA of 29 891 nucleotides which code for 9860 amino acids.^62^ This is enclosed by a nucleocapsid protein, and a lipid membrane through which membrane and envelope proteins protrude, as well as the larger spike glycoproteins (Fig. 12). The spike protein is a primary target for vaccine development.^63^

Clay minerals can bind to virus particles and have antiviral activity. For example, kaolin has anti-hepatitis C virus activity in Huh-7 (liver tumour) cells. Hepatitis C virus (HCV) is a 55–65 nm, enveloped, single-stranded RNA virus.^70^ The nature of the mineral surface (coating) is important for binding. Reovirus type 3 and coliphage T1, for example, do not share common adsorption sites on kaolinite and montmorillonite.^71^ Compounds in growth media (*e.g.* fetal bovine serum, amino acids) in which the reovirus was maintained, blocked adsorption of coliphage T1 to kaolinite.^71^ Silica monolithic HPLC columns can be used for the separation of viruses.^72,73^

### 4.1. Antibacterial and antiviral bismuth oligomers

#### 4.1.1. Bismuth citrate oligomers

**Fig. 13:**
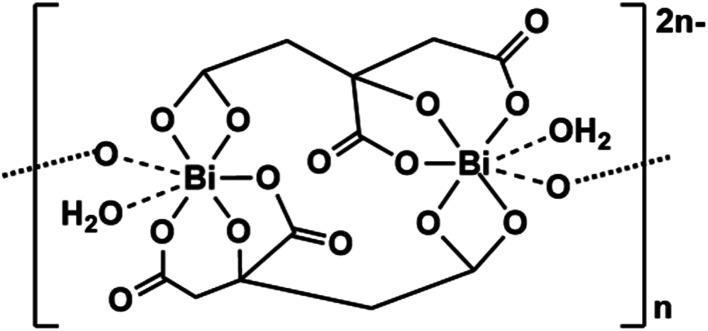
Structure of bismuth citrate.^75^

Aqua Bi(iii) ions are highly acidic (p*K*_a_*ca.* 3) and complexes such as bismuth subcitrate have long been used as antacids.^74^ They have complicated layer structures based on a tetra-deprotonated citrate forming a strong Bi(iii) alkoxide bond, and bonds to the three carboxylates, which bridge two Bi(iii) ions forming a dimer(Fig. 13). The [Bi(cit)_2_Bi]^2−^ dimers with *e.g.* K^+^ or NH_4_^+^ counter cations, further associate with neighbouring dimers, forming an insoluble colloid (colloidal bismuth citrate, CBS). These 2D sheet and 3D polymer structures can bind to the surfaces of cells and tissues.^75^

CBS can be solubilised using the antiulcer drug ranitidine [*N*,*N*-dimethyl-5-(3-nitromethylene-7-thia-2,4-diazaoctyl)furan-2-methanamine] as the counter cation.^76^ Ranitidine bismuth citrate (RBC, the drug Tritec) is used to treat ulcers. The antimicrobial activity of Bi(iii) means that RBC can kill the bacterium *Helicobacter pylori*, a Gram-negative bacterium with 4–6 characteristic flagella, 2.5–4.0 μm in length and 0.5–1.0 μm in width, which can prevent ulcers from healing.^77,78^ Furthermore, RBC also suppresses SARS-CoV-2 replication, relieving virus-associated pneumonia in a hamster model, whilst demonstrating inhibition towards the ATPase and DNA-unwinding of the SARS-CoV-2 helicase by irreversible displacement of Zn(ii) by Bi(iii) *in vitro*.^79^

### 4.2. Antiviral and antibacterial polyoxometallates

**Fig. 14:**
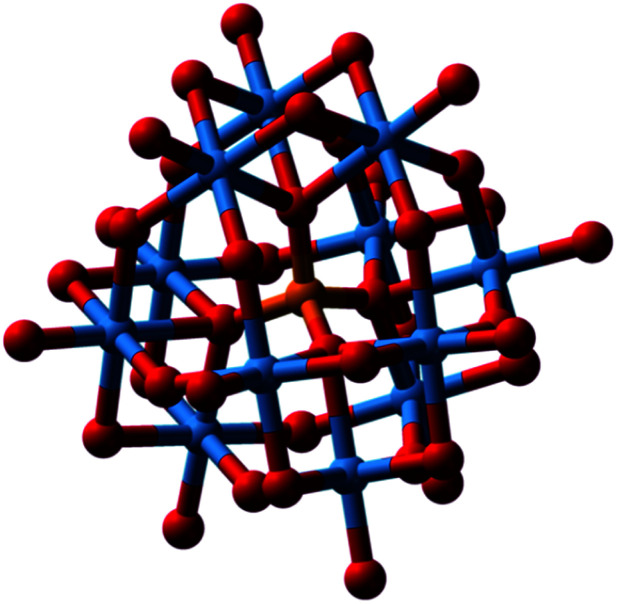
Phosphotungstate anion [PW_12_O_40_]^3−^. O, red; P, orange; W, blue. A central tetrahedral PO_4_ surrounded by 12 octahedral W(vi)O_6_, with bridging and terminal surface oxygens. This is the major component of phosphotungstic acid at pH 1. It is used in histology for staining of cells; it binds to fibrin, collagen, and connective tissues. Reproduced from https://en.wikipedia.org/wiki/Phosphotungstic_acid with permission from author (Benjah-bmm27).

There is much interest in the anticancer, antimicrobial and especially antiviral activity of polyoxometallates (POMs).^80^ There are two major types of POMs, isopoly- and heteropoly-oxoanions, with the general formulae [M_*m*_O_*y*_]^*n*−^ and [X_*x*_M_*m*_O_*y*_]^*n*−^, respectively, where M is often W(vi), Mo(iv) or V(v), and X the central heteroatom, which can be almost any other element. The phosphotungstate anion [PW_12_O_40_]^3−^ (structure first elucidated by Keggin in 1933), for example, consists of a framework of twelve octahedral tungsten oxyanions surrounding a central phosphate group (Fig. 14).^81^ Much of the early biological data have been summarised by Rhule *et al.*^82^

The antiviral activity of POMs was first reported nearly 50 years ago.^83^ Early work focussed on polytungstosilicates. HPA-23, a polytungstoantimoniate [NaSb_9_W_21_O_86_]^18−^ entered clinical trials for the treatment of AIDS, caused by the human immunodeficiency virus, HIV.^84^ However, efficacy *in vivo* was poor and there were toxic side effects. POM antiviral activity varies considerably with the structural class, composition, size and charge of the POM, as well as the virus, viral strain and the cell line used for screening. With the wide variations in compositions of POMs that are accessible, it might be possible to engineer the surface for optimum recognition of the surfaces of specific virus particles. Several recent studies have identified POM candidates for clinical trials.^85,86^ The Keggin-structured polyoxometalate H_3_[PW_12_O_40_] (POM-12, Fig. 14) exhibits micromolar antiviral activity towards Zika, dengue and Japanese encephalitis viruses, and dramatically inhibits their infection ability.^86^ The studies by Li *et al.* suggest that Nb(v) POM Cs_2_K_4_Na[SiW_9_Nb_3_O_40_]·H_2_O exhibits sufficient activity against hepatitis B to warrant clinical trials.^85^ POMs can bind to and inhibit a large number of proteins and enzymes *in vitro*, such as a cationic pocket in HIV-1 protease,^87^ but such targets are yet to be verified *in vivo*.^88^

POMs exhibit promising antibacterial activity.^89^ An early report of antibacterial activity suggested synergism with conventional antibiotics. An aged mixture of tungstate and phosphate, named ‘Factor T’, greatly enhanced the antibacterial effect of β-lactam antibiotics in Gram-positive methicillin-resistant *Staphylococcus aureus* (MRSA) strains.^90^ POM activity depends on composition, shape, and size, but in the case of medium-size polyoxotungstates (POTs, charge > −12 and number of addenda atoms ≤22) the activity correlates with the total net charge.^91^

POMs exhibiting the highest activity towards *Helicobacter pylori* are mostly Keggin-type POTs, polyoxovanadotungstates and large highly negatively-charged POMs, whereas in the case of *Streptococcus pneumoniae*, the most active POMs were ascribed to be polyoxovanadates, especially decavanadate, which was also very active against other bacteria.

Applications of inorganic POMs in medicine are currently limited by their toxicity and the lack of detailed knowledge about their mode of action. Many POMs are thermodynamically and kinetically unstable in physiological media and degrade into a mixture of products. Cation-uptake and exchange in POM and POM-based compounds have been categorized as: (i) POMs as inorganic crown ethers and cryptands, (ii) POM-based ionic solids as cation-exchangers, and (iii) reduction-induced cation-uptake in POM-based ionic solids, based on redox-activity and multi-electron transfer occurring reversibly in multiple steps.^92^ This speciation problem is a challenge for future investigations. Not only the intact POM, but each constituent of a POM may play a role in its activity.

### 4.3. Aluminium adjuvants boost potency of vaccines

Aluminium adjuvants have been added to billions of doses of vaccines for over 90 years, and are administered to millions of people annually.^93^ However, the mechanism by which they boost the effectiveness of vaccines is very poorly understood.^94^ It has been suggested that aluminium adjuvants enhance the delivery of the co-adsorbed antigens to dendritic cells, as well as increasing antigen presentation.^95^ The importance of this application is highlighted by the current (2020) urgency of developing an effective vaccine against the pandemic caused by SARS-CoV-2.

**Fig. 15:**
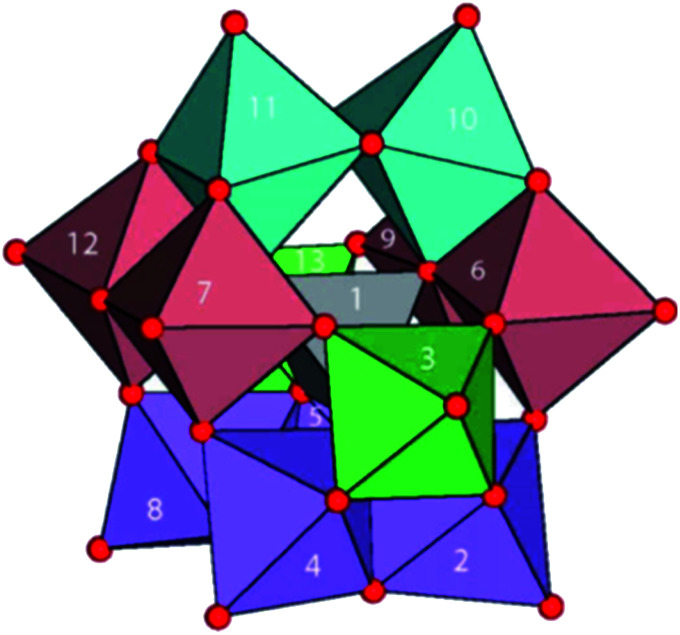
Structure of [Al_13_O_4_(OH)_24_(H_2_O)_12_]^7+^ in its sulfate salt. A central Al(O)_4_ unit surrounded by four trimeric groups of edge-shared Al(O)_6_ octahedra. Red, O; H atoms omitted. Reprinted with permission from *Inorg. Chem.* 2016, **55**, 12270–12280. Copyright 2016 American Chemical Society.^97^

Aquated Al(iii) ions [Al(H_2_O)_6_]^3+^ are highly acidic (p*K*_a_*ca.* 5). Thus, hydroxido and oxido species can readily form and aggregate into oligomers since these are good bridging ligands. Pentameric aluminium complexes, such as [Al_5_(OH)_12_]^3+^ and [Al_5_(OH)_13_]^2+^, and tridecameric “Keggin” cation [AlO_4_Al_12_(OH)_24_(H_2_O)_12_]^7+^, readily form, even at low aluminium concentrations (down to 20 mM).^96^ [Al_13_O_4_(OH)_24_(H_2_O)_12_]^7+^ (Fig. 15) is a γ-Al_13_ Keggin cluster formed during hydrolysis and by aggregation of natural and synthetic Al-oxyhydroxides, and is very well characterized.^97^ Moreover, ligand exchange reactions are relatively slow on Al(iii),^98^ with aging of polymeric species and formation of larger polynuclear complexes taking place on timescales of hours to days.^99^

Typical insoluble adjuvants are Al(O)OH (*e.g.* Alhydrogel®) and Al(OH)_*x*_(PO_4_)_*y*_ (*e.g.* Adju-Phos®), aggregates of 10–50 nm-sized particles. The surface adsorption properties of these nanoparticles towards antigens and protein components of the vaccines are probably key to their adjuvant activity.^93^

## 5. Protein–mineral interfaces

The interactions of living cells and biomolecules with mineral surfaces can be of several types: non-covalent binding, *e.g.* electrostatic (ionic), hydrophobic/van der Waals, H-bonds, and covalent, *e.g.* coordination bonding. Interactions between cells and minerals are likely to be multipoint because of the repeating nature of the mineral structure. Some cells, *e.g.* bacteria, utilise excreted reducing or oxidizing agents so as to erode the surface and utilise mineral components for energy and as nutrients. They may also excrete a metal chelating agent, such as a siderophore which binds to iron and delivers it into the cell. Some microorganisms are involved in the weathering of minerals in rocks, soils and sediments, and can also mediate the formation of biominerals.^100^

There is structural evidence for the interaction between hydroxo/oxo minerals and the serum protein transferrin (Tf), which transports iron to cells, and its bacterial analogue ferric ion binding protein (Fbp), which transports iron across the periplasm of Gram-negative bacteria.^101^ Fbp is a virulence factor in microorganisms such as *Neisseria gonorrhoeae*.^102^ The involvement of transferrin in the transport of a range of metal ions is potentially important for understanding the transport of metallodrugs, diagnostic agents, radioisotopes, and toxic metal ions.^101^ Iron-loaded Fe(iii)_2_-Tf is recognised by a specific receptor on cells and transported into cells in endosomes, where the Fe(iii) is released at *ca.* pH 5.6, and the receptor is recycled.

The 679-amino acid, 80 kDa glycoprotein Tf, present in serum at *ca.* 30 μM, binds Fe(iii) much more tightly than Fe(ii) in a cleft in each of its 2 lobes, which are connected by a short peptide linker. The Fe(iii) ligands are 2 Tyr, 1 Asp and 1 His, as well as an important so-called ‘synergistic anion’, which in native Tf is carbonate. The open cleft closes once the carbonate is bound. Binding of Tf to a mineral surface can change the shape of the protein. On a freshly cleaved mica (KAl_2_(AlSi_3_O_10_)(OH)_2_) surface, Tf contracts from its average ‘native’ (X-ray crystal structure) of *ca.* 8 nm to *ca.* 2.5 nm.^103^

**Fig. 16:**
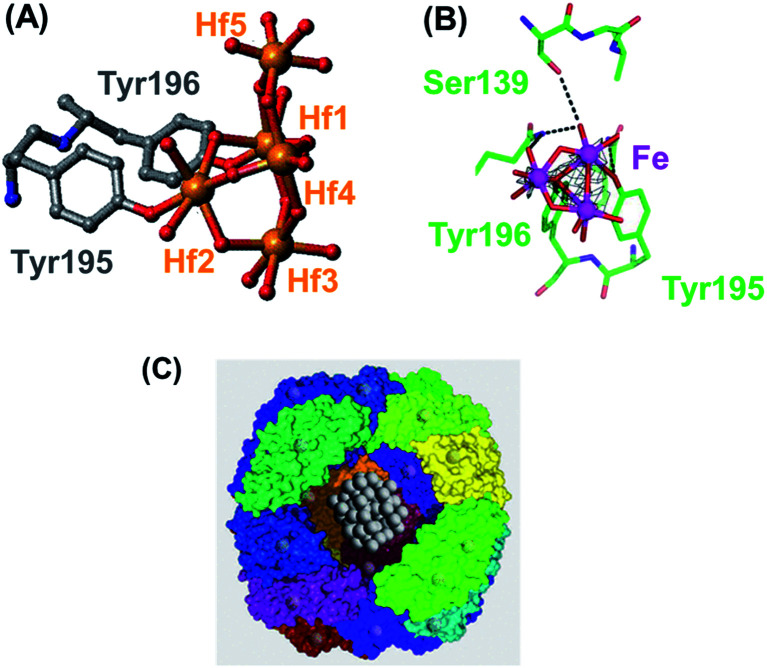
(A) A pentanuclear oxo-Hf(iv) cluster in the open interdomain cleft of bacterial transferrin (Fbp), bound to the protein by two tyrosinate side-chains, Tyr195 and Tyr196 (adapted from Alexeev *et al.*, 2003).^104^ This cluster can also be capped by phosphate. (B) Trinuclear Fe_3_O_4_ oxo-iron cluster in Fe_3_–Fbp. The phenolate oxygen of Tyr196 is a central μ^3^-bridging oxygen and Tyr195 is monodentate (adapted from Zhu *et al.*, 2003).^105^ (C) The ferrihydrite-like (Fe(O)OH) mineral core in the Fe(iii) storage protein ferritin. Part of the 24 subunit protein shell has been cut away to show the ferrihydrite inside. Courtesy of Allen D. Elster, https://MRIquestions.com.

Fbp (34 kDa, 309 amino acids) has a structure similar to one of the two lobes of serum transferrin. Fbp also uses 2 Tyr (tyrosinate) ligands to bind Fe(iii), and one His, but now also Glu and a synergistic anion such as phosphate. The protein effectively integrates metal-binding groups in the side-chains of amino acids into mineral structures as illustrated by the X-ray crystal structures of Fbp containing fragments of the hafnium(iv) mineral HfO_2_ and (oxy/hydroxy)iron minerals. Fbp can be crystallized with tri- and pentanuclear oxo-Hf_3_ and oxo-Hf_5_ clusters which resemble fragments of the mineral HfO_2_, in an open binding cleft.^104^ These clusters are anchored by binding to tyrosinate oxygens (Fig. 16), which can also be capped by phosphate. Similarly, bound trinuclear Fe_3_O_4_ (Fe_3_O_13_) oxo-Fe(iii)clusters can be characterized in X-ray structures.^105^ Both serum and bacterial transferrins can bind a range of other 3+ and 4+ multinuclear complexes strongly, such as Zr(iv) and Ti(iv).^106^

## 6. Neurochemistry – minerals in the brain

There is a wide variety of metals in the brain, including weakly binding and fast-moving Na^+^ and K^+^ ions. Brain neurons transmit signals using a flow of Na^+^ and K^+^ ions through metal-specific ion channels, producing an electrical spike (action potential). The channels include a Na^+^/K^+^ pump which uses the energy of one ATP to exchange 3 intracellular Na^+^ ions for 2 extracellular K^+^ ions.^107^ Mg^2+^ and Ca^2+^ also have specific membrane pumps, but are less mobile and involved in stronger protein and enzyme binding.^108^ Notably, Mg^2+^ at millimolar concentrations also binds to DNA and ATP, and has a major function in blocking the Ca^2+^ channel in the (*N*-methyl-*d*-aspartate) NMDA receptor.^109^ NMDA is an agonist for glutamate, the neurotransmitter which normally acts at that receptor.

**Fig. 17:**
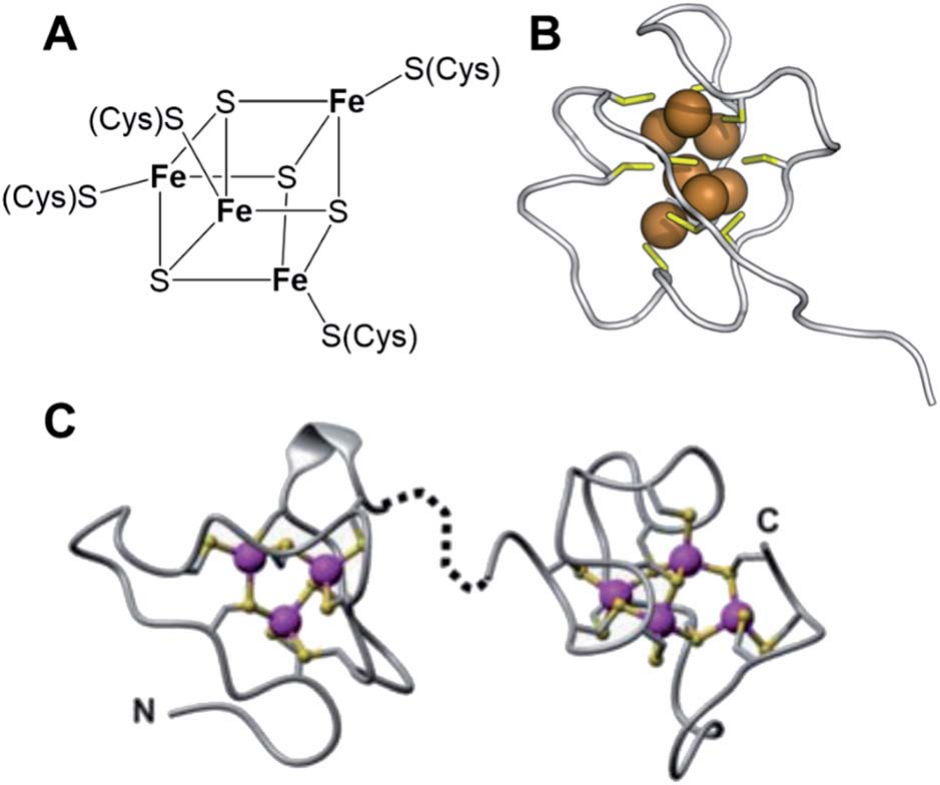
(A) An Fe_4_S_4_ cluster in a ferredoxin. The protein environment around the cluster can modulate the redox potential (the Fe(ii)/Fe(iii) ratio). (B) A cluster of Cu(i) ions (brown) bridged by cysteine sulfurs (yellow) in a metallothionein (yeast, PDB: 1AQS). (C) Cd(or Zn)_3_Cys_9_ and Cd(orZn)_4_Cys_11_ clusters in (rat liver) metallothionein (PDB 1mrt and 2mrt).^111^ (B) and (C) reproduced from ref. 111 with permission from the Royal Society of Chemistry, copyright 2010.^111^

Manganese, iron, cobalt, copper, zinc and molybdenum also play important roles in the brain. Manganese enzymes include superoxide dismutase and glutamine synthetase, and Cu(i) and Zn(ii) form clusters in brain metallothionein MT3.^110^ Metallothioneins are small cysteine-rich proteins (typically one third Cys from a total of *ca.* 60 amino acids) which are ubiquitous in cells and contain clusters of Zn(ii), Cd(ii) and Cu(i) bridged by thiolate sulfurs (Fig. 17).^111^

Multi-iron centres are ubiquitous in the brain and elsewhere in the body. Iron-sulfur clusters, with sulfide and cysteine thiolate coordination *e.g.* Fe_4_S_4_ cubane structures consisting of four Fe(ii)/Fe(iii) ions bridged by sulfides and further coordinated to cysteine thiolate sulfurs to give 4-coordinate Fe (Fig. 17), serve as redox centres in many proteins and biochemical pathways.^112^

Nearly 30 years ago, strongly magnetic particles of magnetite (Fe_3_O_4_) were detected in the human brain, 5 million single-domain crystals per gram in most tissues.^113,114^ Some of these particles have been described as pollutant magnetite particles <∼200 nm in diameter, which can enter the brain directly *via* the olfactory bulb. Iron accumulation and oxidative stress appear to be early events in the development of Alzheimer's disease. Analysis of brain tissue by Scanning Transmission X-ray Microscopy (STXM) reveals mixed oxidation states of neuronal iron deposits associated with neuromelanin clusters in Parkinson's disease substantia nigra.^115^

Iron is stored in cells as the mineral ferrihydrite, Fe(iii)(O)OH, with some incorporation of phosphate. Storage of *ca.* 4500 Fe(iii) ions occurs inside the shell of the protein ferritin, which consists of 24 *ca*. 20 kDa subunits, a diameter of *ca.* 10 nm (Fig. 16).^116^ Fe(ii) is taken up through channels between the subunits and is oxidised to Fe(iii) before it enters the mineral core. The co-aggregation of peptide β-amyloid (Aβ) and ferritin resulting in the conversion of ferritin's inert ferric core into more reactive low-oxidation-state iron phases may play a role in Alzheimer's disease.^117^

X-ray spectromicroscopy and X-ray magnetic circular dichroism studies have revealed that amyloid plaque cores (APC) are associated with diffuse iron, and dense iron deposits incorporating ferrous iron, as well as the mixed-valence iron oxide magnetite (Fe_3_O_4_). Intriguingly, evidence consistent with the presence of zero-oxidation state iron has been reported.^118^ Moreover, calcium deposits were also observed within APC, including evidence for mineralization – plaque calcification and calcium carbonate deposition.^118^

## 7. Conclusions and outlook

Inorganic chemistry (mineral chemistry) was once widely considered to be relevant only to non-living organisms, in contrast to the chemistry of carbon, organic chemistry. However, we now know that life would not exist without inorganic chemistry and the essentiality of at least 18 elements in addition to carbon. Here we have highlighted the need to widen the exploration of the biological and medicinal chemistry of minerals because they offer potential for the discovery of the causes of disease and scope for the design of novel therapies, as well as applications in biotechnology.

However, the chemistry and biochemistry of minerals is dynamic and very diverse. They have inherently heterogeneous structures, relating to both their surfaces and their bulk composition. Their compositions can change with time by metal cation and anion exchange, on wide timescales. Even polymorphs having the same chemical composition, but different packing arrangements, can have different biological properties (*e.g.* calcium carbonate crystals in the inner ear, Fig. 6).

Although the study of minerals presents significant challenges, recent advances in technology promise to open up new understanding of their formation, reactions, and properties, including studies in native biological environments. These include synchrotron X-ray methods (*e.g.* nano-focussed X-ray fluorescence), laser-ablation-ICP-MS, electron microscopy (*e.g.* atomic resolution TEM), mass spectrometry (*e.g.* nano-SIMS), and radio-tracing/imaging using beta- and gamma-emitting radioisotopes.

Some minerals have very high lattice energies and are relatively inert in terms of their overall frameworks. Examples include clinically-approved lanthanum carbonate for the lowering of phosphate levels in the body, which act by carbonate–phosphate exchange, and zirconium cyclosilicate for controlling potassium levels by cation exchange in pores. There is much scope for the design of other such therapeutic minerals. In contrast, some minerals are highly toxic, including the silicate asbestos, and nickel subsulfide. The reasons for their toxicity are not fully understood, but may be related to both their surface properties and exchange/release of the bulk components. The release of redox-active transition metals, such as iron and copper, from minerals can be a source of toxicity, especially iron release from asbestos (chrysotile).^119^

The calcium phosphate mineral hydroxyapatite plays vital roles in bones and teeth, but also its surface properties can be controlled to provide widely used chromatographic supports for separation of DNA and RNA oligomers, proteins, and viruses *via* multipoint recognition. Design of such mineral particles for therapy should be further explored.

We have discussed an example of a molecular mechanism for the recognition of minerals by proteins, oxo/hydroxo-metal complexes in the iron binding cleft of the iron transport proteins serum transferrin and bacterial ferric ion-binding protein. Such binding has been demonstrated for higher oxidation state (acidic) metal ions such as Ti(iv), Zr(iv) and Hf(iv), as well as Fe(iii). Deprotonated phenolates from protein tyrosines can anchor mineral fragments by forming coordination bonds. Investigations are now needed as to whether such interactions can occur in intact biological systems, and if so, what are the consequences?

Understanding the roles of metals in the brain promises to shed new light on a range of poorly understood conditions such as senile dementia, Parkinson's, and Alzheimer's diseases. Particularly intriguing is the discovery of nanomineralization in the brain, and even deposits of low oxidation state iron, metallic Fe(0). It will be challenging to elucidate the chemistry and biochemistry of such species, especially in intact brain tissue.

Traditional Chinese Medicine (TCM) has long led the way in exploring the potential for use of minerals in medicine. Now modern molecular pharmacology can use all the techniques and methods currently available, including genomics and proteomics (metallomics) and state-of-the-art metal speciation and imaging to reveal new roles for metal minerals in disease and therapy, which is exciting for future research.

## Conflicts of interest

There are no conflicts to declare.

## Supplementary Material

## References

[cit1] Chellan P., Sadler P. J. (2015). Philos. Trans. R. Soc., A.

[cit2] Mann S., Ozin G. A. (1996). Nature.

[cit3] Li M., Schnablegger H., Mann S. (1999). Nature.

[cit4] Totura A. L., Bavari S. (2019). Expert Opin. Drug Discovery.

[cit5] Yu W., Foster H. D., Zhang T. (1995). J. Orthomol. Med..

[cit6] Marie B., Marie A., Jackson D. J., Dubost L., Degnan B. M., Milet C., Marin F. (2010). Proteome Sci..

[cit7] Mann K., Cerveau N., Gummich M., Fritz M., Mann M., Jackson D. J. (2018). Proteome Sci..

[cit8] Liu J., Shi J. Z., Yu L. M., Goyer R. A., Waalkes M. P. (2008). Exp. Biol. Med..

[cit9] Tian J. Z., Liang A. H., Zhu X. X., Zhao Y., Yi Y., Li C. Y., Han J. Y. (2019). World J. Tradit. Chin. Med..

[cit10] Wang Y., Xu C. H., Wang P., Sun S. Q., Chen J. B., Li J., Chen T., Wang J. B. (2011). Spectrochim. Acta, Part A.

[cit11] Zhou J., Qu F. (2009). Afr. J. Tradit., Complementary Altern. Med..

[cit12] Flora S. J. S. , Arsenic: Chemistry, Occurrence, and Exposure in Handbook of Arsenic Toxicology, ed S. J. S. Flora, Elsevier Inc., 2015, Ch. 1

[cit13] Tinggi U., Sadler R., Ng J., Noller B., Seawright A. (2016). Regul. Toxicol. Pharmacol..

[cit14] Man S., Gao W., Wei C., Liu C. (2012). Phyther. Res..

[cit15] Jain A., Ong S. P., Hautier G., Chen W., Richards W. D., Dacek S., Cholia S., Gunter D., Skinner D., Ceder G., Persson K. A. (2013). APL Mater..

[cit16] Zhu N. , Han S., Yang C., Qu J., Sun Z., Liu W. and Zhang X., Element-tracing of mineral matters in Dendrobium officinale using ICP-MS and multivariate analysis. Springerplus; 5(1):979 10.1186/s40064-016-2618-2 PMC4932019 27429889

[cit17] Barry N. P. E., Pitto-Barry A., Sanchez A. M., Dove A. P., Procter R. J., Soldevila-Barreda J. J., Kirby N., Hands-Portman I., Smith C. J., O'Reilly R. K., Beanland R., Sadler P. J. (2014). Nat. Commun..

[cit18] Pitto-Barry A., Geraki K., Horbury M. D., Stavros V. G., Mosselmans J. F. W., Walton R. I., Sadler P. J., Barry N. P. E. (2017). Chem. Commun..

[cit19] Pitto-Barry A., Perdigao L. M. A., Walker M., Lawrence J., Costantini G., Sadler P. J., Barry N. P. E. (2015). Dalton Trans..

[cit20] Pitto-Barry A., Sadler P. J., Barry N. P. E. (2016). Chem. Commun..

[cit21] Lermyte F., Zhang W. Y., Brooks J., Huband S., Collingwood J. F., Lees M. R., Rayman M. P., Sadler P. J. (2020). Food Funct..

[cit22] Giljohann D., Seferos D., Daniel W., Massich M., Patel P., Mirkin C. (2014). Angew. Chem., Int. Ed. Engl..

[cit23] Chithrani B. D., Ghazani A. A., Chan W. C. W. (2006). Nano Lett..

[cit24] Vines J. B., Yoon J. H., Ryu N. E., Lim D. J., Park H. (2019). Front. Chem..

[cit25] Lerner U. H. (2012). Semin. Orthod..

[cit26] Yu L., Rowe D. W., Perera I. P., Zhang J., Suib S. L., Xin X., Wei M. (2020). ACS Appl. Mater. Interfaces.

[cit27] Li C., Zhao H., Liu Z., McMahon C. (2014). Int. J. Biochem. Cell Biol..

[cit28] Palmer L. C., Newcomb C. J., Kaltz S. R., Spoerke E. D., Stupp S. I. (2008). Chem. Rev..

[cit29] Leroy N., Bres E., Jones D. B., Downes S. (2001). Eur. Cell. Mater..

[cit30] Cummings L. J., Snyder M. A., Brisack K. (2009). Methods Enzymol..

[cit31] Itoh D., Yoshimoto N., Yamamoto S. (2018). Curr. Protein Pept. Sci..

[cit32] Fadrosh D. W., Andrews-Pfannkoch C., Williamson S. J. (2011). J. Visualized Exp..

[cit33] Andrews-Pfannkoch C., Fadrosh D. W., Thorpe J., Williamson S. J. (2010). Appl. Environ. Microbiol..

[cit34] Mann S., Parker S. B., Ross M. D., Skarnulis A. J., Williams R. J. (1983). Proc. R. Soc. London, Ser. B.

[cit35] Dai Y., Zou H., Zhu H., Zhou X., Song Y., Shi Z., Sheng Y. (2017). CrystEngComm.

[cit36] Lyubchenko Y. L., Shlyakhtenko L. S., Ando T. (2011). Methods.

[cit37] Fu Y., Romero M. J., Salassa L., Cheng X., Habtemariam A., Clarkson G. J., Prokes I., Rodger A., Costantini G., Sadler P. J. (2016). Angew. Chem., Int. Ed..

[cit38] Effects, Committee Practices, Board , Asbestos: Selected cancers, 2006, 2006, pp. 1–327. 10.17226/11665

[cit39] Gordon R. E., Fitzgerald S., Millette J. (2014). Int. J. Occup. Environ. Health.

[cit40] Ristić M., Czakó-Nagy I., Musić S., Vértes A. (2011). J. Mol. Struct..

[cit41] Artali R., Del Pra A., Foresti E., Lesci I. G., Roveri N., Sabatino P. (2008). J. R. Soc., Interface.

[cit42] Hostýnek J. J. (2002). Arch. Dermatol. Res..

[cit43] Muñoz A., Costa M. (2012). Toxicol. Appl. Pharmacol..

[cit44] Li Q., Li L., Wu P., Xu N., Wang L., Li M., Dai A., Amine K., Mai L., Lu J. (2019). Adv. Energy Mater..

[cit45] Drozdov A. P., Kong P. P., Minkov V. S., Besedin S. P., Kuzovnikov M. A., Mozaffari S., Balicas L., Balakirev F. F., Graf D. E., Prakapenka V. B., Greenberg E., Knyazev D. A., Tkacz M., Eremets M. I. (2019). Nature.

[cit46] Lo Faro M., Zignani S. C., Aricò A. S. (2020). Materials.

[cit47] Swainston Harrison T., Scott L. J. (2004). Drugs.

[cit48] Finn W. F. (2005). Therapy.

[cit49] Shinn D. B., Eick H. A. (1968). Inorg. Chem..

[cit50] Murrer B. A. and Powell N. A., Pharmaceutical composition containing selected lanthanum carbonate hydrates, *US Pat.* Application US5968976, 1998

[cit51] Van Mater H. L. , Zirconium compound deoderant and antiperspirant, *US Pat.*US2498514A, United States Pat. Off., 1946

[cit52] Urban J., Fergus D. J., Savage A. M., Ehlers M., Menninger H. L., Dunn R. R., Horvath J. E. (2016). PeerJ.

[cit53] Bretagne A., Cotot F., Arnaud-Roux M., Sztucki M., Cabane B., Galey J. B. (2017). Soft Matter.

[cit54] Linder K. E., Krawczynski M. A., Laskey D. (2016). Pharmacotherapy.

[cit55] Stavros F., Yang A., Leon A., Nuttall M., Rasmussen H. S. (2014). PLoS One.

[cit56] Yan Y., Zhang J., Ren L., Tang C. (2016). Chem. Soc. Rev..

[cit57] Whittell G. R., Hager M. D., Schubert U. S., Manners I. (2011). Nat. Mater..

[cit58] Shi Y., van der Meel R., Chen X., Lammers T. (2020). Theranostics.

[cit59] Larnaudie S. C., Brendel J. C., Romero-Canelón I., Sanchez-Cano C., Catrouillet S., Sanchis J., Coverdale J. P. C., Song J. I., Habtemariam A., Sadler P. J., Jolliffe K. A., Perrier S. (2018). Biomacromolecules.

[cit60] Graham S. P., El-Sharif H. F., Hussain S., Fruengel R., McLean R. K., Hawes P. C., Sullivan M. V., Reddy S. M. (2019). Front. Bioeng. Biotechnol..

[cit61] Lodish H. , Berk A. and Zipursky S. L., in Molecular Cell Biology, W. H. Freeman, New York, 4th edn, 2000

[cit62] Chan J. F., Kok K. (2020). Emerging Microbes Infect..

[cit63] Watanabe Y., Allen J. D., Wrapp D., McLellan J. S., Crispin M. (2020). Science.

[cit64] Zhu N., Zhang D., Wang W., Li X., Yang B., Song J., Zhao X., Huang B., Shi W., Lu R., Niu P., Zhan F., Ma X., Wang D., Xu W., Wu G., Gao G. F., Tan W. (2020). N. Engl. J. Med..

[cit65] Wang Q., Zhang Y., Wu L., Niu S., Song C., Zhang Z., Lu G., Qiao C., Hu Y., Yuen K. Y., Wang Q., Zhou H., Yan J., Qi J. (2020). Cell.

[cit66] Shang J., Ye G., Shi K., Wan Y., Luo C., Aihara H., Geng Q., Auerbach A., Li F. (2020). Nature.

[cit67] Walls A. C., Park Y. J., Tortorici M. A., Wall A., McGuire A. T., Veesler D. (2020). Cell.

[cit68] Astuti I., Ysrafil (2020). Diabetes Metab. Syndr. Clin. Res. Rev..

[cit69] Eckert A. and Higgins D., Public Health Image Library (PHIL), accessed 22 December 2020, https://phil.cdc.gov/Details.aspx?pid=23312

[cit70] Ali L., Idrees M., Ali M., Hussain A., Rehman I. U., Ali A., Iqbal S. A., Kamel E. H. (2014). BMC Res. Notes.

[cit71] Lipson S. M., Stotzky G. (1985). Can. J. Microbiol..

[cit72] Silva R. J. S., Mendes J. P., Carrondo M. J. T., Marques P. M., Peixoto C. (2020). Methods Mol. Biol..

[cit73] Kramberger P., Urbas L., Štrancar A. (2015). Hum. Vaccines Immunother..

[cit74] Asato E., Driessen W. L., de Graaff R. A. G., Hulsbergen F. B., Reedijk J. (1991). Inorg. Chem..

[cit75] Li W., Jin L., Zhu N., Hou X., Deng F., Sun H. (2003). J. Am. Chem. Soc..

[cit76] Sadler P. J., Sun H. (1995). J. Chem. Soc., Dalton Trans..

[cit77] Wang R., Lai T. P., Gao P., Zhang H., Ho P. L., Woo P. C. Y., Ma G., Kao R. Y. T., Li H., Sun H. (2018). Nat. Commun..

[cit78] Li H., Wang R., Sun H. (2019). Acc. Chem. Res..

[cit79] Yuan S., Wang R., Chan J. F. W., Zhang A. J., Cheng T., Chik K. K. H., Ye Z. W., Wang S., Lee A. C. Y., Jin L., Li H., Jin D. Y., Yuen K. Y., Sun H. (2020). Nat. Microbiol..

[cit80] Yamase T. , Polyoxometalates active against tumors, viruses, and bacteria ed. Müller W., Wang X. and Schröder H., in Biomedical Inorganic Polymers. Progress in Molecular and Subcellular Biology, Springer, Berlin. 2013, vol 54 10.1007/978-3-642-41004-8_4 PMC7122307 24420711

[cit81] Keggin J. F. (1933). Nature.

[cit82] Rhule J. T., Hill C. L., Judd D. A., Schinazi R. F. (1998). Chem. Rev..

[cit83] Raynaud M., Chermann J. C., Plata F., Jasmin C., Mathé G., Acad C. R. (1971). C. R. Seances Acad. Sci., Ser. D.

[cit84] Rozenbaum W., Dormont D., Spire B., Vilmer E., Gentilini M., Griscelli C., Montagnier L., Barre-Sinoussi F., Chermann J. C. (1985). Lancet.

[cit85] Li Q., Zhang H., Qi Y., Wang J., Li J., Niu J. (2019). Drug Dev. Res..

[cit86] Qi Y., Han L., Qi Y., Jin X., Zhang B., Niu J., Zhong J., Xu Y. (2020). Antiviral Res..

[cit87] Judd D. A., Nettles J. H., Nevins N., Snyder J. P., Liotta D. C., Tang J., Ermolieff J., Schinazi R. F., Hill C. L. (2001). J. Am. Chem. Soc..

[cit88] Zhao M., Chen X., Chi G., Shuai D., Wang L., Chen B., Li J. (2020). Inorg. Chem. Front..

[cit89] Bijelic A., Aureliano M., Rompel A. (2018). Chem. Commun..

[cit90] Tajima Y., Nagasawa Z., Tadano J. (1993). Microbiol. Immunol..

[cit91] Gumerova N. I., Al-Sayed E., Krivosudský L., Ĉipĉić-Paljetak H., Verbanac D., Rompel A. (2018). Front. Chem..

[cit92] Uchida S. (2019). Chem. Sci..

[cit93] HogenEsch H., O'Hagan D. T., Fox C. B. (2018). npj Vaccines.

[cit94] Fox C. B. and Walker J. M., Vaccine Adjuvants: Methods and Protocols, Humana Press, 2016. vol. 1494

[cit95] Shardlow E., Mold M., Exley C. (2018). Allergy, Asthma, Clin. Immunol..

[cit96] Akitt J. W., Elders J. M. (1988). J. Chem. Soc., Dalton Trans..

[cit97] Phillips B. L., Ohlin C. A., Vaughn J., Woerner W., Smart S., Subramanyam R., Pan L. (2016). Inorg. Chem..

[cit98] Richens D. T. (2005). Chem. Rev..

[cit99] Bi S., Wang C., Cao Q., Zhang C. (2004). Coord. Chem. Rev..

[cit100] Gadd G. M. (2010). Microbiology.

[cit101] Sun H., Li H., Sadler P. J. (1999). Chem. Rev..

[cit102] Guo M., Harvey I., Yang W., Coghill L., Campopiano D. J., Parkinson J. A., MacGillivray R. T. A., Harris W. R., Sadler P. J. (2003). J. Biol. Chem..

[cit103] Booyjzsen C., Scarff C. A., Moreton B., Portman I., Scrivens J. H., Costantini G., Sadler P. J. (2012). Biochim. Biophys. Acta, Gen. Subj..

[cit104] Alexeev D., Zhu H., Guo M., Zhong W., Hunter D. J. B., Yang W., Campopiano D. J., Sadler P. J. (2003). Nat. Struct. Biol..

[cit105] Zhu H., Alexeev D., Hunter D. J. B., Campopiano D. J., Sadler P. J. (2003). Biochem. J..

[cit106] Zhong W., Alexeev D., Harvey I., Guo M., Hunter D. J. B., Zhu H., Campopiano D. J., Sadler P. J. (2004). Angew. Chem., Int. Ed..

[cit107] Morth J. P., Pedersen B. P., Toustrup-Jensen M. S., Sørensen T. L. M., Petersen J., Andersen J. P., Vilsen B., Nissen P. (2007). Nature.

[cit108] Olesen C., Picard M., Winther A. M. L., Gyrup C., Morth J. P., Oxvig C., Møller J. V., Nissen P. (2007). Nature.

[cit109] Blanke M. L. and VanDongen A. M. J., in Biology of the NMDA Receptor, ed. A. Van Dongen, CRC Press/Taylor & Francis, Boca Raton, FL, 2009 21204416

[cit110] Vašák M., Meloni G. (2017). Int. J. Mol. Sci..

[cit111] Blindauer C. A., Leszczyszyn O. I. (2010). Nat. Prod. Rep..

[cit112] Braymer J. J., Lill R. (2017). J. Biol. Chem..

[cit113] Kirschvink J. L., Kobayashi-Kirschvink A., Woodford B. J. (1992). Proc. Natl. Acad. Sci. U. S. A..

[cit114] Maher B. A., Ahmed I. A. M., Karloukovski V., MacLaren D. A., Foulds P. G., Allsop D., Mann D. M. A., Torres-Jardón R., Calderon-Garciduenas L. (2016). Proc. Natl. Acad. Sci. U. S. A..

[cit115] Brooks J., Everett J., Lermyte F., Tjendana Tjhin V., Sadler P. J., Telling N., Collingwood J. F. (2020). J. Trace Elem. Med. Biol..

[cit116] Chiou B., Connor J. R. (2018). Pharmaceuticals.

[cit117] Everett J., Brooks J., Lermyte F., O'Connor P. B., Sadler P. J., Dobson J., Collingwood J. F., Telling N. D. (2020). Sci. Rep..

[cit118] Everett J., Collingwood J. F., Tjendana-Tjhin V., Brooks J., Lermyte F., Plascencia-Villa G., Hands-Portman I., Dobson J., Perry G., Telling N. D. (2018). Nanoscale.

[cit119] Mohanty S. K., Gonneau C., Salamatipour A., Pietrofesa R. A., Casper B., Christofidou-Solomidou M., Willenbring J. K. (2018). J. Hazard. Mater..

